# ADHD: Current Concepts and Treatments in Children and Adolescents

**DOI:** 10.1055/s-0040-1701658

**Published:** 2020-06-19

**Authors:** Renate Drechsler, Silvia Brem, Daniel Brandeis, Edna Grünblatt, Gregor Berger, Susanne Walitza

**Affiliations:** 1 Department of Child and Adolescent Psychiatry and Psychotherapy, University Hospital of Psychiatry, University of Zurich, Zurich, Switzerland; 2 Neuroscience Center Zurich, Swiss Federal Institute of Technology and University of Zurich, Zurich, Switzerland; 3 Department of Child and Adolescent Psychiatry and Psychotherapy, Central Institute of Mental Health, Medical Faculty Mannheim/Heidelberg University, Mannheim, Germany; 4 Zurich Center for Integrative Human Physiology, University of Zurich, Zurich, Switzerland

**Keywords:** ADHD, etiology, treatment

## Abstract

Attention deficit hyperactivity disorder (ADHD) is among the most frequent disorders within child and adolescent psychiatry, with a prevalence of over 5%. Nosological systems, such as the Diagnostic and Statistical Manual of Mental Disorders, 5th edition (DSM-5) and the International Classification of Diseases, editions 10 and 11 (ICD-10/11) continue to define ADHD according to behavioral criteria, based on observation and on informant reports. Despite an overwhelming body of research on ADHD over the last 10 to 20 years, valid neurobiological markers or other objective criteria that may lead to unequivocal diagnostic classification are still lacking. On the contrary, the concept of ADHD seems to have become broader and more heterogeneous. Thus, the diagnosis and treatment of ADHD are still challenging for clinicians, necessitating increased reliance on their expertise and experience. The first part of this review presents an overview of the current definitions of the disorder (DSM-5, ICD-10/11). Furthermore, it discusses more controversial aspects of the construct of ADHD, including the dimensional versus categorical approach, alternative ADHD constructs, and aspects pertaining to epidemiology and prevalence. The second part focuses on comorbidities, on the difficulty of distinguishing between “primary” and “secondary” ADHD for purposes of differential diagnosis, and on clinical diagnostic procedures. In the third and most prominent part, an overview of current neurobiological concepts of ADHD is given, including neuropsychological and neurophysiological researches and summaries of current neuroimaging and genetic studies. Finally, treatment options are reviewed, including a discussion of multimodal, pharmacological, and nonpharmacological interventions and their evidence base.

## Introduction

With a prevalence of over 5%, attention deficit hyperactivity disorder (ADHD) is one of the most frequent disorders within child and adolescent psychiatry. Despite an overwhelming body of research, approximately 20,000 publications have been referenced in PubMed during the past 10 years, assessment and treatment continue to present a challenge for clinicians. ADHD is characterized by the heterogeneity of presentations, which may take opposite forms, by frequent and variable comorbidities and an overlap with other disorders, and by the context-dependency of symptoms, which may or may not become apparent during clinical examination. While the neurobiological and genetic underpinnings of the disorder are beyond dispute, biomarkers or other objective criteria, which could lead to an automatic algorithm for the reliable identification of ADHD in an individual within clinical practice, are still lacking. In contrast to what one might expect after years of intense research, ADHD criteria defined by nosological systems, such as the Diagnostic and Statistical Manual of Mental Disorders, 5th edition (DSM-5) and the International Classification of Diseases, editions 10 and 11 (ICD-10/11) have not become narrower and more specific. Rather, they have become broader, for example, encompassing wider age ranges, thus placing more emphasis on the specialist's expertise and experience. 1 2 3

## Definitions and Phenomenology

### ADHD According to the DSM-5 and ICD-10/11

ADHD is defined as a neurodevelopmental disorder. Its diagnostic classification is based on the observation of behavioral symptoms. ADHD according to the DSM-5 continues to be a diagnosis of exclusion and should not be diagnosed if the behavioral symptoms can be better explained by other mental disorders (e.g., psychotic disorder, mood or anxiety disorder, personality disorder, substance intoxication, or withdrawal). 1 However, comorbidity with other mental disorders is common.

In the DSM-5, the defining symptoms of ADHD are divided into symptoms of inattention (11 symptoms) and hyperactivity/impulsivity (9 symptoms). 1 The former differentiation between subtypes in the DSM-IV proved to be unstable and to depend on the situational context, on informants, or on maturation, and was therefore replaced by “presentations.” 4 Thus, the DSM-5 distinguishes between different presentations of ADHD: predominantly inattentive (6 or more out of 11 symptoms present), predominantly hyperactive/impulsive (6 or more out of 9 symptoms present), and combined presentation (both criteria fulfilled), as well as a partial remission category. Symptoms have to be present in two or more settings before the age of 12 years for at least 6 months and have to reduce or impair social, academic, or occupational functioning. In adolescents over 17 years and in adults, five symptoms per dimension need to be present for diagnosis. 1 In adults, the use of validated instruments like the Wender Utah rating scale is recommended. 5

In contrast, the ICD-10 classification distinguishes between hyperkinetic disorder of childhood (with at least six symptoms of inattention and six symptoms of hyperactivity/impulsivity, present before the age of 6 years) and hyperkinetic conduct disorder, a combination of ADHD symptoms and symptoms of oppositional defiant and conduct disorders (CD). 3 In the ICD-11 (online release from June 2018, printed release expected 2022), the latter category has been dropped, as has the precise age limit (“onset during the developmental period, typically early to mid-childhood”). Moreover, the ICD-11 distinguishes five ADHD subcategories, which match those of the DSM-5: ADHD combined presentation, ADHD predominantly inattentive presentation, ADHD predominantly hyperactive/impulsive presentation and two residual categories, ADHD other specified and ADHD nonspecified presentation. For diagnosis, behavioral symptoms need to be outside the limits of normal variation expected for the individual's age and level of intellectual functioning. 2

### Overlapping Constructs: Sluggish Cognitive Tempo and Emotional Dysregulation

Sluggish cognitive tempo (SCT) is a clinical construct characterized by low energy, sleepiness, and absent-mindedness, and is estimated to occur in 39 to 59% of (adult) individuals with ADHD. 6 7 The question of whether SCT might constitute a feature of ADHD or a separate construct that overlaps with ADHD inattention symptoms is unresolved. 8 While current studies indicate that SCT might be distinct and independent from hyperactivity/impulsivity, as well as from inattention dimensions, it remains uncertain whether it should be considered as a separate disorder. 8 9 Twin studies have revealed a certain overlap between SCT and ADHD, especially with regard to inattention symptoms, but SCT seems to be more strongly related to nonshared environmental factors. 10

Emotion dysregulation is another associated feature that has been discussed as a possible core component of childhood ADHD, although it is not included in the DSM-5 criteria. Deficient emotion regulation is more typically part of the symptom definition of other psychopathological disorders, such as oppositional defiant disorder (ODD), CD, or disruptive mood dysregulation disorder (DSM-5; for children up to 8 years). 11 However, an estimated 50 to 75% of children with ADHD also present symptoms of emotion dysregulation, for example, anger, irritability, low tolerance for frustration, and outbursts, or sometimes express inappropriate positive emotions. The presence of these symptoms increases the risk for further comorbidities, such as ODD and also for anxiety disorders. 12 13 For adult ADHD, emotional irritability is a defining symptom according to the Wender Utah criteria, and has been confirmed as a primary ADHD symptom by several studies (e.g., Hirsch et al). 5 14 15

Whether emotion dysregulation is inherent to ADHD, applies to a subgroup with combined symptoms and a singular neurobiological pathway, or is comorbid with but independent of ADHD, is still a matter of debate (for a description of these three models; Shaw et al 13 ). Faraone et al 12 distinguished three ADHD prototypes with regard to deficient emotion regulation: ADHD prototype 1 with high-emotional impulsivity and deficient self-regulation, prototype 2 with low-emotional impulsivity and deficient self-regulation, and prototype 3 with high-emotional impulsivity and effective self-regulation. All three prototypes are characterized by an inappropriate intensity of emotional response. While prototypes 1 and 3 build up their responses very quickly, prototype 2 is slower to respond but experiences higher subjective emotional upheaval than is overtly shown in the behavior. Prototypes 1 and 2 both need more time to calm down compared with prototype 3 in which emotional self-regulation capacities are intact.

### Dimensional versus Categorical Nature of ADHD

Recent research on subthreshold ADHD argues in favor of a dimensional rather than categorical understanding of the ADHD construct, as its core symptoms and comorbid features are dimensionally distributed in the population. 16 17 18 Subthreshold ADHD is common in the population, with an estimated prevalence of approximately 10%. 19 According to Biederman and colleagues, clinically referred children with subthreshold ADHD symptoms show a similar amount of functional deficits and comorbid symptoms to those with full ADHD, but tend to come from higher social-class families with fewer family conflicts, to have fewer perinatal complications, and to be older and female (for the latter two, a confound with DSM-IV criteria cannot be excluded). 20

### Temperament and Personality Approaches to ADHD

Another approach which is in accordance with a dimensional concept is to analyze ADHD and categorize subtypes according to temperament/personality traits (for a review and the different concepts of temperament see Gomez and Corr 21 ). Temperament/personality traits are usually defined as neurobiologically based constitutional tendencies, which determine how the individual searches for or reacts to external stimulation and regulates emotion and activity. While temperament traits per se are not pathological, extreme variations or specific combinations of traits may lead to pathological behavior. This approach has been investigated in several studies by Martel and colleagues and Nigg, 22 23 24 who employed a temperament model comprising three empirically derived domains 25 26 : (1) negative affect, such as tendencies to react with anger, frustration, or fear; (2) positive affect or surgency which includes overall activity, expression of happiness, and interest in novelty; and (3) effortful control which is related to self-regulation and the control of action. The latter domain shows a strong overlap with the concept of executive function. 27 In a community sample, early temperamental traits, especially effortful control and activity level, were found to potentially predict later ADHD. 28 Karalunas et al 29 30 distinguished three temperament profiles in a sample of children with ADHD: one with normal emotional functioning; one with high surgency, characterized by high levels of positive approach-motivated behaviors and a high–activity level; and one with high negative (“irritable”) affect, with the latter showing the strongest, albeit only moderate stability over 2 years. Irritability was not reducible to comorbidity with ODD or CD and was interpreted as an ADHD subgroup characteristic with predictive validity for an unfavorable outcome. These ADHD temperament types were distinguished by resting-state and peripheral physiological characteristics as measured by functional magnetic resonance imaging (fMRI). 29

### Epidemiology and Prevalence

While ADHD seems to be a phenomenon that is encountered worldwide, 31 prevalence rates and reported changes in prevalence are highly variable, depending on country and regions, method, and sample. 32 A meta-analysis by Polanczyk et al 32 yielded a worldwide prevalence rate of 5.8% in children and adolescents. 33 In an update published 6 years later, the authors did not find evidence for an increase in prevalence over a time span of 30 years. Other meta-analyses reported slightly higher (e.g., 7.2%) 34 or lower prevalence rates, which seems to be attributable to the different criteria adopted for defining ADHD. Prevalence rates in children and adolescents represent averaged values across the full age range, but peak prevalence may be much higher in certain age groups, for example, 13% in 9-year-old boys. 35 Universal ADHD prevalence in adults is estimated to lie at 2.8%, with higher rates in high-income (3.6%) than in low-income (1.4%) countries. 36 True prevalence rates (also called community prevalence, e.g., Sayal et al 37 ) should be based on population-based representative health surveys, that is, the actual base rate of ADHD in the population, in contrast to the administrative base rate, which is related to clinical data collection (Taylor 38 ). Recent reports on the increase in ADHD rates usually refer to administrative rates, drawn from health insurance companies, from the number of clinical referrals for ADHD, 39 clinical case identification estimates, or from the percentage of children taking stimulant medication (prescription data). Changes in these rates may be influenced by increased awareness, destigmatization, modifications in the defining criteria of ADHD, or altered medical practice. According to a recent U.S. health survey on children and adolescents (4–17 years), in which parents had to indicate whether their child had ever been diagnosed with ADHD, the percentage of diagnoses increased from 6.1% in 1997 to 10.2% in 2016. 40 A representative Danish survey based on health registry, data collected from 1995 to 2010 reported that ADHD incidence rates increased by a factor of approximately 12 (for individuals aged 4–65 years) during this period. Moreover, the gender ratio decreased from 7.5:1 to 3:1 at early school age and from 8.1:1 to 1.6:1 in adolescents in the same time frame, 41 42 probably indicating an improved awareness of ADHD symptoms in girls. In other countries, it is assumed that girls are still underdiagnosed. 38

Population register data show that the use of stimulants for ADHD has increased considerably worldwide. 43 In most countries, an increase in stimulant medication use has been observed in children since the 1990s (e.g., United Kingdom from 0.15% in 1992 to 5.1% in 2012/2013), 44 45 but in some European countries, stimulant prescription rates for children and adolescents have remained stable or decreased over the last 5 to 10 years (e.g., Germany). 35 In the United States, the prescription of methylphenidate peaked in 2012 and has since been slightly decreasing, while the use of amphetamines continues to rise. 46

## Comorbidity, Differential Diagnosis, and Clinical Assessment

### Comorbidity

ADHD is characterized by frequent comorbidity and overlap with other neurodevelopmental and mental disorders of childhood and adolescence. The most frequent comorbidities are learning disorders (reading disorders: 15–50%, 4 dyscalculia: 5–30%, 47 autism spectrum disorder, which since the DSM-5 is no longer viewed as an exclusion criterion for ADHD diagnosis: 70–85%, 48 49 tic/Tourette's disorder and obsessive compulsive disorder: 20%, and 5%, 50 developmental coordination disorder: 30–50%, 51 depression and anxiety disorders: 0–45%, 52 53 and ODD and CD: 27–55% 54 ). ADHD increases the risk of substance misuse disorders 1.5-fold (2.4-fold for smoking) and problematic media use 9.3-fold in adolescence 55 56 and increases the risk of becoming obese 1.23-fold for adolescent girls. 57 58 59 It is also associated with different forms of dysregulated eating in children and adolescents. Enuresis occurs in approximately 17% of children with ADHD, 60 and sleep disorders in 25 to 70%. 61 Frequent neurological comorbidities of ADHD include migraine (about thrice more frequent in ADHD than in typically developing [TD] children) 62 63 64 and epilepsy (2.3 to thrice more frequent in ADHD than in TD children). 65 66 The risk of coexisting ADHD being seen as a comorbid condition and not the primary diagnosis is considerably enhanced in many childhood disorders of different origins. For example, the rate of comorbid ADHD is estimated at 15 to 40% 67 68 in children with reading disorders and at 26 to 41% 69 70 in children with mild intellectual dysfunction. While comorbidity in neurodevelopmental disorders may arise from a certain genetic overlap (see details under genetic associations), ADHD symptoms are also present in several disorders with well-known and circumscribed genetic defects, normally not related to ADHD (e.g., neurofibromatosis, Turner's syndrome, and Noonan's syndrome) 71 or disorders with nongenetic causes, such as traumatic brain injuries, pre-, peri- or postnatal stroke, or syndromes due to toxic agents, such as fetal alcohol syndrome. Comorbid ADHD is estimated in 20 to 50% of children with epilepsy, 72 73 in 43% of children with fetal alcohol syndrome, 74 and in 40% of children with neurofibromatosis I. 75 ADHD is three times more frequent in preterm-born children than in children born at term and four times more frequent in extremely preterm-born children. 76

#### Differential Diagnosis, Primary and Secondary ADHD

A range of medical and psychiatric conditions show symptoms that are also present in primary ADHD. The most important medical conditions which are known to “mimic” ADHD and need to be excluded during the diagnostic process are epilepsy (especially absence epilepsy and rolandic epilepsy), thyroid disorders, sleep disorder, drug interaction, anemia, and leukodystrophy. 77 78 The most important psychiatric conditions to be excluded are learning disorder, anxiety disorders, and affective disorders, while an adverse home environment also needs to be excluded.

However, the picture is complex, as many differential diagnoses may also occur as comorbidities. For instance, bipolar disorder, which is frequently diagnosed in children and adolescents in the United States but not in Europe, is considered as a differential diagnosis to ADHD, but ADHD has also been found to be a comorbidity of bipolar disorder in 21 to 98% of cases. 79 Similarly, absence epilepsy is a differential diagnosis of ADHD but is also considered to be a frequent comorbidity, occurring in 30 to 60% of children with absence epilepsy. 80 The prevalence of the ADHD phenotype in benign childhood epilepsy with centrotemporal spikes (rolandic epilepsy) lies at 64 to 65%, 81 and is possibly related to the occurrence of febrile convulsions. 82 The literature often does not draw a clear distinction between an ADHD phenotype, which includes all types of etiologies and causes, and a yet to be specified developmental ADHD “genotype.” Some authors use terms, such as “idiopathic” ADHD, 83 “primary,” or “genotypic” ADHD, 84 in contrast to ADHD of circumscribed origin other than developmental, the latter being referred to as ADHD “phenotype,” or “phenocopy,” 85 or “ADHD-like.” 86 “Secondary ADHD” usually refers to newly acquired ADHD symptoms arising after a known event or incident, for example, a head trauma or stroke. After early childhood stroke, the ADHD phenotype occurs in 13 to 20% of cases, and after pediatric traumatic brain injury, ADHD symptoms are observed in 15 to 20% of children. 87 Having ADHD considerably increases the risk of suffering a traumatic brain injury, 88 89 90 and most studies on secondary ADHD after traumatic brain injury control for or compare with premorbid ADHD (e.g., Ornstein et al 91 ). Whether and to what extent “phenotypic” and “genotypic” ADHD need to be distinguished on a phenomenological level is not clear. It is possible that shared neurobiological mechanisms will prevail and that genetic vulnerability and epigenetic factors may play a role in both types. For example, James et al 86 compared neurophysiological markers in two groups of adolescents with ADHD, one born very preterm and the other born at term. While the authors found very similar ADHD-specific markers in the two groups, some additional deficits only emerged in the preterm group, indicating more severe impairment. Other examples are rare genetic diseases with known genetic defects, which are often comorbid with ADHD. One may ask whether, for example, ADHD in Turner's syndrome should be considered as a rare genetic ADHD variant and count as genotypic ADHD, or whether it results from a different genetic etiology, with the status of an ADHD phenotype.

### Clinical Diagnostic Procedure

Clinical assessment in children should mainly be based on a clinical interview with parents, including an exploration of the problems, the detailed developmental history of the child including medical or psychiatric antecedents, information on family functioning, peer relationships, and school history. According to the guidelines of the National Institute for Health and Care Excellence (NICE) in the United Kingdom, this may also include information on the mental health of the parents and the family's economic situation. The child's mental state should be assessed, possibly using a standardized semistructured clinical interview containing ADHD assessments (e.g., Kiddie Schedule for Affective Disorders and Schizophrenia Present and Lifetime version, DSM-5) 92 93 and by observer reports. The exploration should cover behavioral difficulties and strengths in several life contexts, for example, school, peer relationships, and leisure time. The use of informant rating scales, such as Conners' Rating Scales, 3rd edition, 94 or the Strengths and Difficulties Questionnaire 95 may be useful, but diagnosis should not be solely based on rating scales (NICE, AWFM ADHD). 96 97 A further interview should be conducted with the child or adolescent to gain a picture of the patient's perspective on current problems, needs, and goals, even though self-reports are considered less reliable for diagnosis. Information should also be obtained from the school, for example, by face-to-face or telephone contact with the teacher and, if possible, by direct school-based observation. A medical examination should be performed to exclude somatic causes for the behavioral symptoms and to gain an impression of the general physical condition of the patient. Current guidelines do not recommend including objective test procedures (intelligence and neuropsychological tests), neuroimaging, or neurophysiological measures in routine ADHD assessment but do suggest their use as additional tools when questions about cognitive functions, academic problems, coexisting abnormalities in electroencephalography (EEG), or unrecognized neurological conditions arise. After completion of the information gathering, the NICE guidelines recommend a period of “watchful waiting” for up to 10 weeks before delivering a formal diagnosis of ADHD. A younger age of the diagnosed child relative to his/her classmates has to be mentioned as one of the many pitfalls in the assessment of ADHD. It has been shown that the youngest children in a class have the highest probability of being diagnosed with ADHD and of being medicated with stimulants. 98

There is consensus that the diagnosis of ADHD requires a specialist, that is, a child psychiatrist, a pediatrician, or other appropriately qualified health care professionals with training and expertise in diagnosing ADHD. 97

## Current Neurobiological and Neuropsychological Concepts

### Neuropsychology

#### Neuropsychological Pathways and Subgroups

ADHD is related to multiple underlying neurobiological pathways and heterogeneous neuropsychological (NP) profiles. Twenty-five years ago, ADHD was characterized as a disorder of inhibitory self-control, 54 and an early dual pathway model distinguished between an inhibitory/executive function pathway and a motivational/delay aversion pathway (also called “cool” and “hot” executive function pathways in later publications), which are related to distinct neurobiological networks. 99 100 101 Still, the two systems may also interact. 102

Since then, other pathways have been added, such as time processing, 103 but a definitive number of possible pathways is difficult to define. For example, Coghill and colleagues 104 differentiated six cognitive factors in children with ADHD (working memory, inhibition, delay aversion, decision-making, timing, and response time variability) derived from seven subtests of the Cambridge neuropsychological test automated battery. Attempts to empirically classify patients into subgroups with selective performance profiles departing from comprehensive NP data collection were inconclusive. For example, using delay aversion, working memory, and response-time tasks, Lambek and colleagues 105 expected to differentiate corresponding performance profile subgroups in children with ADHD. However, their analysis resulted in subgroups differentiated by the severity of impairments, and not by selective profiles. Other empirical studies using latent profile or cluster analysis of NP tasks in large ADHD samples have differentiated three 106 107 or four 108 NP profile groups, which all included children with ADHD, as well as TD children, differing in severity but not in the type of profile. This might indicate that the identified NP deficit profiles were not ADHD-specific, but rather reflected characteristic distributions of NP performances, which are also present in the general population, with extreme values in children with ADHD. Some other empirical studies in the search for subgroups, however, identified ADHD-specific performance profiles (“poor cognitive control,” 109 “with attentional lapses and fast processing speed” 110 ), among other profiles being shared with TD controls. Obviously, divergent results regarding subgrouping may also be related to differing compilations of tested domains, consequently leading to a limited comparability of these studies.

#### Which Neuropsychological Functions are Impaired in ADHD and When?

A meta-analysis conducted in 2005 identified consistent executive function deficits with moderate effect sizes in children with ADHD in terms of response inhibition, vigilance, working memory, and planning. 67 Since then, a vast number of studies on NP deficits in children with ADHD compared with TD controls have been published. A recent meta-analysis included 34 meta-analyses on neurocognitive profiles in ADHD (all ages) published until 2016, referring to 12 neurocognitive domains. 111 The authors found that 96% of all standardized mean differences were positive in favor of the control group. Unweighted effect sizes ranged from 0.35 (set shifting) to 0.66 (reaction time variability). Weighted mean effect sizes above 0.50 were found for working memory (0.54), reaction time variability (0.53), response inhibition (0.52), intelligence/achievement (0.51), and planning/organization (0.51). Effects were larger in children and adolescents than in adults. The other domains comprised vigilance, set shifting, selective attention, reaction time, fluency, decision making, and memory.

Nearly every neuropsychological domain has been found to be significantly impaired in ADHD compared with TD controls, though effect sizes are often small. This includes, for example, altered perception (e.g., increased odor sensitivity 112 ; altered sensory profile 113 ; impaired yellow/blue color perception, e.g., Banaschewski et al, 114 for review, see Fuermaier et al 115 ), emotional tasks (e.g., facial affect discrimination), 116 social tasks (e.g., Marton et al 117 ), communication, 118 and memory. 119 Several of the described impairments may be related to deficient top-down cognitive control and strategic deficits, 120 121 122 but there is also evidence for basic processing deficits. 123

#### Neuropsychological Deficits as Mediators of Gene-Behavior Relations

A vast amount of research has been devoted to the search for neuropsychological endophenotypes (or intermediate phenotypes) for ADHD, that is, neurobiologically based impairments of NP performance characteristic of the disorder that may also be found in nonaffected close relatives. ADHD neuropsychological endophenotypes are assumed to mediate genetic risk from common genetic variants. 124 So far, deficits in working memory, reaction-time variability, inhibition, time processing, response preparation, arousal regulation, and others have been identified as probable endophenotypes for ADHD. 124 125 126 127 Genetic studies indicate an association of an ADHD-specific polygenetic general risk score (i.e., the total number of genetic variants that may be associated with ADHD, mostly related to dopaminergic transmission) with working memory deficits and arousal/alertness, 124 or with a lower intelligence quotient (IQ) and working memory deficits, 128 respectively. More specifically, a link of ADHD-specific variants of *DAT1* genes with inattention and hyperactivity symptoms seems to be mediated by inhibitory control deficits. 129

#### Individual Cognitive Profiles and the Relevance of Cognitive Testing for the Clinical Assessment

Heterogeneity is found with regard to profiles, as well as with regard to the severity of cognitive impairment in individuals with ADHD, as measured by standardized tests. ADHD does not necessarily come with impaired neuropsychological test performance: about one-third of children with ADHD will not present any clinically relevant impairment, while another one-third shows unstable or partial clinical impairment, and about another one-third performs below average in NP tests. The classic concept of NP impairment, which assumes relative stability over time, possibly does not apply to NP deficits observed in ADHD, or only to a lesser extent. For the larger part, the manifestation of performance deficits may depend on contextual factors, 130 such as reward, or specifically its timing, amount, and nature, or on energetic factors, 131 for example, the rate of stimulus presentation or the activation provided by the task.

Many studies have shown that behavioral ratings of ADHD symptoms or questionnaires on executive function deficits are not, or at best weakly, correlated with NP test performance, even when both target the same NP domain. 132 133 In consequence, questionnaires on executive functioning are not an appropriate replacement for neuropsychological testing. Likewise, ADHD symptom rating scales do not predict results of objective attention or executive function tests and vice versa. Although mild intellectual disability and low IQ are more typically associated with the disorder, ADHD can be encountered across the entire IQ spectrum, including highly gifted children. 134 Therefore, an intelligence test should be part of the diagnostic procedure, but is not mandatory according to ADHD guidelines. In some children, intellectual difficulties and not ADHD may be the underlying cause for ADHD-like behaviors, while in other children with ADHD, academic underachievement despite a high IQ may be present.

It has been argued that symptoms defining ADHD may be understood as dimensional markers of several disorders belonging to an ADHD spectrum and, in consequence, the diagnosis of these behavioral symptoms should be the starting point for a more in-depth diagnosis rather than the endpoint. 135 This should include the cognitive performance profile. The ADHD behavioral phenotype predicts neither NP impairment nor intellectual achievement in the individual case, and objective testing is the only way to obtain an accurate picture of the child's cognitive performance under standardized conditions. Its goal is not ADHD classification, but rather to obtain the best possible understanding of the relation between cognitive functioning and behavioral symptoms for a given patient, to establish an individually tailored treatment plan.

### Neurophysiology

Neurophysiological methods like EEG, magnetoencephalography, and event-related potentials (ERPs) as task-locked EEG averages capture brain functions in ADHD at high (ms) temporal resolution. The approach covers both fast and slow neural processes and oscillations, and clarifies the type and timing of brain activity altered in ADHD at rest and in tasks. It reveals neural precursors, as well as correlates, and consequences of ADHD behavior. 136 Neurophysiological and particularly EEG measures also have a long and controversial history as potential biomarkers of ADHD. Current evidence clarifies how multiple pathways and deficits are involved in ADHD at the group level, but recent attempts toward individual clinical translation have also revealed considerable heterogeneity, which does not yet support a clinical application for diagnostic uses or treatment personalization, as explained below.

#### Resting Electroencephalography

The EEG is dominated by oscillations in frequency bands ranging from slow δ (<4 Hz) and θ (4–7 Hz) via α (8–12 Hz) to faster β (13–30 Hz) and γ (30–100 Hz) band activity. The spectral profile reflects maturation and arousal, with slow frequencies dominating during early childhood and slow-wave sleep. Source models can link scalp topography to brain sources and distributed networks.

Initial studies suggested a robust link between ADHD diagnosis and resting EEG markers of reduced attention, hypoarousal, or immaturity, such as increased θ and an increased θ/β ratio (TBR). However, more recent studies, 137 138 some with large samples, 139 140 failed to replicate a consistent TBR increase in ADHD. Instead, the results indicated heterogeneous θ and β power deviations in ADHD not explained by ADHD subtype and psychiatric comorbidity. 141 A cluster analysis of EEG in children with ADHD also revealed considerable heterogeneity regarding θ excess and β attenuation in ADHD. While several clusters with EEG patterns linked to underarousal and immaturity could be identified, only three of the five EEG clusters (60% of the cases with ADHD) had increased θ. 139 Several recent θ and TBR studies that no longer found TBR association with ADHD diagnosis still replicated the reliable age effects, 137 138 142 confirming the high quality of these studies. Increasing sleepiness in adolescents, 143 or shorter EEG recordings, may have reduced the sensitivity to time effects and state regulation deficits in ADHD, 136 144 potentially contributing to these replication failures. Also, conceptualizing TBR as a marker of inattention or maturational lag may be too simple, since θ activity can also reflect concentration, cognitive effort, and activation. 145 146

During sleep, stage profiles reveal no consistent deviations in ADHD, but the slow-wave sleep topography is altered. In particular, frontal slow waves are reduced, leading to a more posterior topography as observed also in younger children. 147 This delayed frontalization can be interpreted as a maturational delay in ADHD, in line with a cluster of resting EEG, changes in task related ERPs during response inhibition, 148 and structural magnetic resonance imaging (MRI) findings. 149

#### Task Related Event-Related Potentials

Task-related processing measures, particularly ERPs, have critically advanced our understanding of ADHD through their high-time resolution, which can separate intact and compromised brain functions. ERPs have revealed impairments during preparation, attention, inhibition, action control, as well as error, and reward processing, with partly distinct networks but often present during different phases of the same task. In youth and adults with ADHD, the attentional and inhibitory P3 components and the preparatory contingent negative variation (CNV) component are most consistently affected, but state regulation and error or reward processing are also compromised. 136 150 Activity during preparation, attention, or inhibition is typically weaker and more variable but not delayed. This often occurs in task phases without visible behavior and precedes the compromised performance. Familial and genetic factors also modulate these markers of attention and control. Some impairment is also observed in nonaffected siblings or in parents without ADHD, 151 152 and genetic correlates often implicate the dopamine system. 125 Some ERP changes, like the attenuated CNV during preparation, remain stable throughout maturation, and are also markers of persistent ADHD, while other markers, such as the inhibition related P3, remain attenuated despite clinical remission. 148 153

Overall, the ERP results confirm attentional, cognitive, and motivational, rather than sensory or motor impairments in ADHD, in line with current psychological and neurobiological models. However, different ERP studies hardly used the same tests and measures, so valid statements regarding classification accuracy and effect size are particularly difficult, 154 and there is an urgent need for meta-analyses regarding the different ERPs.

#### Clinical Translation

Despite published failures to replicate robust TBR based classification of ADHD, a TBR-based EEG test was recently approved by the U.S. Food and Drug Administration to assist ADHD diagnosis. 155 Although not promoted as a stand-alone test, children with suspected ADHD, and increased TBR were claimed to likely meet full diagnostic criteria for ADHD; while children with suspected ADHD but no TBR increase should undergo further testing, as they were likely to have other disorders better explaining ADHD symptoms (see also DSM-5 exclusionary criterion E).

This multistage diagnostic approach could possibly identify a homogeneous neurophysiological subgroup, but it omits critical elements of careful, guideline-based ADHD diagnostics. Reliability and predictive value of the TBR remain untested, and the increasing evidence for poor validity of TBR renders it unsuitable for stand-alone ADHD diagnosis. Accordingly, the use of TBR as a diagnostic aid was broadly criticized. 156 157

In sum, the recent literature suggests that neither TBR nor other single EEG or ERP markers are sufficient to diagnose ADHD and are not recommended for clinical routine use, in line with the increasing evidence for heterogeneity in ADHD.

Combining measures across time, frequency, and tasks or states into multivariate patterns may better characterize ADHD. The potential of such approaches is evident in improved classification using machine-learning algorithms based on combinations of EEG measures 142 or EEG and ERP measures. 138 158 However, claims of high-classification accuracies up to 95% (e.g., Mueller et al 158 ) require further independent replication and validation with larger samples, and plausible mapping to neural systems and mechanisms. Modern pattern classification is particularly sensitive to uncontrolled sample characteristics and needs validation through independent large samples. 159

Focusing on EEG-based prediction rather than diagnosis may hold more promise for clinical translation, and may utilize the EEG heterogeneity in clinical ADHD samples. For example, early studies on predicting stimulant response suggested that children with altered wave activity, in particular increased TBR, θ or α slowing, respond well to stimulant medication. However, in recent prospective work with a large sample, TBR was not predictive, and α slowing allowed only limited prediction in a male adolescent subgroup. 160

Predicting response to intense nonpharmacological treatment is of particular interest given the high costs and time requirements. Promising findings have been reported for one neurofeedback study, where α EEG activity and stronger CNV activity together predicted nearly 30% of the treatment response. 161 Still, the lack of independent validation currently allows no clinical application.

In conclusion, neurophysiological measures have clarified a rich set of distinct impairments but also preserved functions which can also serve as markers of persistence or risk. These markers may also contribute in the classification of psychiatric disorders based on neuromarkers (research domain criteria approach). As potential predictors of treatment outcome they may support precision medicine, and proof-of-concept studies also highlight the potential of multivariate profiling. The findings also demonstrate the challenge with this approach, including notable replication failures, and generalizability of most findings remains to be tested. Neurophysiological markers are not ready to serve as tools or aids to reliably diagnose ADHD, or to personalize ADHD treatment in individual patients.

### Neuroimaging

Modern brain imaging techniques have critically contributed to elucidating the etiology of ADHD. While MRI provides detailed insights into the brain microstructure, such as for example gray matter volume, density, cortical thickness, or white matter integrity, fMRI allows insights into brain functions through activation and connectivity measures with high–spatial resolution.

#### Delayed Maturation and Persistent Alterations in the Brain Microstructure in ADHD

The brain undergoes pronounced developmental alterations in childhood and adolescence. Gray matter volume and cortical thickness show nonlinear inverted **U** -shaped trajectories of maturation with a prepubertal increase followed by a subsequent decrease until adulthood while white matter volume progressively increases throughout adolescence and early adulthood in a rather linear way. 162 163 164 165 Large variations of the maturational curves in different brain regions and subregions suggest that phylogenetically older cortical areas mature earlier than the newer cortical regions. Moreover, brain areas associated with more basic motor or sensory functions mature earlier than areas associated with more complex functions including cognitive control or attention. 163 164 Altered maturation of the cortex for ADHD has been reported for multiple areas and cortical dimensions, 166 167 mainly in the form of delayed developmental trajectories in ADHD but recently also as persistent reductions, particularly in the frontal cortex. 168 Such findings speak for delayed maturation in specific areas rather than a global developmental delay of cortical maturation in ADHD. Microstructural alterations in ADHD have been associated with a decreased intracranial volume 169 and total brain size reduction of around 3 to 5%. 100 168 170 In accordance, increasing ADHD symptoms in the general population correlated negatively with the total brain size. 171 A meta-analysis (Frodl et al) and a recent cross-sectional mega- and meta-analysis (Hoogman et al) indicate that such reductions in brain volume may be due to decreased gray matter volumes in several subcortical structures, such as the accumbens, amygdala, caudate, hippocampus, and putamen but also cortical areas (prefrontal, the parietotemporal cortex) and the cerebellum. 170 172 173 174 175 176 177 Effects sizes of subcortical alterations were highest in children with ADHD and the subcortical structures showed a delayed maturation. 169 Moreover, higher levels of hyperactivity/impulsivity in children were associated with a slower rate of cortical thinning in prefrontal and cingulate regions. 167 178 Differences in brain microstructure have also been reported in a meta-analysis for white matter integrity as measured with diffusion tensor imaging in tracts subserving the frontostriatal-cerebellar circuits. 179 To summarize, diverse neuroanatomical alterations in total brain volume and multiple cortical and subcortical dimensions characterize ADHD. These alterations are most pronounced in childhood and suggest a delayed maturation of specific cortical and subcortical areas along with some persistent reductions in frontal areas in a subgroup of ADHD patients with enduring symptoms into adulthood.

#### Alterations in the Brain Function of Specific Networks in ADHD

Specific functional networks, mainly those involved in inhibition, attention processes, cognitive control, reward processing, working memory, or during rest have been intensively studied in ADHD using fMRI in the past. Alterations have been reported in the corresponding brain networks and the main findings are summarized below.

#### Atypical Resting State Connectivity in Children with ADHD

Resting state examines spontaneous, low frequency fluctuations in the fMRI signal during rest, that is , in absence of any explicit task. 180 Resting state networks describe multiple brain regions for which the fMRI signal is correlated (functionally connected) at rest, but the same networks may coactivate also during task-based fMRI. 181 One important resting state network, the so-called default mode network (DMN), comprises brain areas that show higher activation during wakeful rest and deactivations with increasing attentional demands. 182 183 While the DMN usually shows decreasing activation with increasing attentional demands, the cognitive control network shows an opposite pattern and increases its activation. This inverse correlation of DMN and the cognitive control networks is diminished or absent in children and adults with ADHD and may explain impaired sustained attention through attentional lapses that are mediated by the DMN. 181 184 185 186 In addition, a more diffuse pattern of resting state networks connectivity and a delayed functional network development in children with ADHD have been reported. 187 Finally, atypical connectivity in cognitive and limbic cortico-striato-thalamo-cortical loops of patients with ADHD suggest that the neural substrates may either reside in impaired cognitive network and/or affective, motivational systems. 181

#### Altered Processing of Attention and Inhibition in Fronto-basal Ganglia Circuits in ADHD

Meta-analyses summarizing the findings of functional activation studies report most consistent alterations in brain activation patterns as hypoactivation of the frontoparietal network for executive functions and the ventral attention system for attentional processes in children with ADHD. 188 189 190 More specifically, motor or interference inhibition tasks yielded consistent decreases in a (right lateralized) fronto-basal ganglia network comprising supplementary motor area, anterior cingulate gyrus, left putamen, and right caudate in children with ADHD. 189 190 For tasks targeting attentional processes, decreased activation in a mainly right lateralized dorsolateral fronto-basal ganglia-thalamoparietal network characterized children with ADHD. Depending on the task, hyperactivation can cooccur in partly or distinct cerebellar, cortical, and subcortical regions. 188 189 190

#### Altered Reward Processing and Motivation

Emotion regulation and motivation is mediated by extended orbitomedial and ventromedial frontolimbic networks in the brain. 191 Abnormal sensitivity to reward seems to be an important factor in the etiology of ADHD as suggested by several models of ADHD, 192 193 194 mainly due to a hypofunctioning dopaminergic system. 195 In accordance, impairments in specific signals that indicate violations of expectations, the so called reward prediction errors (RPE), were shown in the medial prefrontal cortex of adolescents with ADHD during a learning task. 196 RPE signals are known to be encoded by the dopaminergic system of the brain, and deficient learning and decision making in ADHD may thus be a consequence of impaired RPE processing. 196 Abnormal activation has also been reported for the ventral striatum during reward anticipation and in other cortical and subcortical structures of the reward circuitry. 197

#### Normalization of Atypical Activation and Brain Structural Measures after Treatment

Stimulant medication and neurofeedback studies have pointed to a certain normalization of dysfunctional activation patterns in critical dorsolateral frontostriatal and orbitofrontostriatal regions along with improvements in ADHD symptoms. 198 199 200 201 Also, brain microstructure, especially the right caudate, has shown some gradual normalization with long-term stimulant treatment. 176 190

To conclude, a wide range of neuroimaging studies reveal relatively consistent functional deficits in ADHD during executive functions, including inhibitory control, working memory, reward processes, and attention regulation but also during rest. Some of these alterations are more persistent, others are specific to children and may thus represent a developmental delay. Specific treatments showed trends toward a normalization of alterations in brain microstructure and functional networks.

### Genetic Associations with ADHD and ADHD Related Traits

From family studies, as well as twin studies, the heritability for ADHD has been estimated to be between 75 upto 90%. 202 Moreover, the heritability was found to be similar in males and females and for inattentive and hyperactive-impulsive components of ADHD. 202 Interestingly, a strong genetic component was also found when the extreme and subthreshold continuous ADHD trait symptoms were assessed in the Swedish twins. 19 Even over the lifespan, adult ADHD was found to demonstrate high heritability that was not affected by shared environmental effects. 203 Recently, structural and functional brain connectivity assessed in families affected by ADHD has been shown to have heritable components associated with ADHD. 204 Similarly, the heritability of ERPs elicited in a Go/No-Go-task measuring response inhibition known to be altered in ADHD, was found to be significantly heritable. 205

In several studies, ADHD-related traits have also shown significant heritability. For example, in two independent, population based studies, significant single nucleotide polymorphism heritability estimates were found for attention-deficit hyperactivity symptoms, externalizing problems, and total problems. 206 In another study, investigating the two opposite ends of ADHD symptoms, low-extreme ADHD traits were significantly associated with shared environmental factors without significant heritability. 207 While on the other hand, high-extreme ADHD traits showed significant heritability without shared environmental influences. 207 A crossdisorder study including 25 brain disorders from genome wide association studies (GWAS) of 265,218 patients and 784,643 controls, including their relationship to 17 phenotypes from 1,191,588 individuals, could demonstrate significant shared heritability. 208 In particular, ADHD shared common risk variants with bipolar disorder, major depressive disorder, schizophrenia, and with migraine. 208 Indeed, in general, population-based twin studies suggest that genetic factors are associated with related-population traits for several psychiatric disorders including ADHD. 209 This suggests that many psychiatric disorders are likely to be a continuous rather than a categorical phenotype.

Though ADHD was found to be highly heritable, the underlying genetic risk factors are still not fully revealed. The current consensus suggests, as in many other psychiatric disorders, a multifactorial polygenic nature of the common disorder. Both common genetic variants studied by hypothesis-driven candidate gene association or by the hypothesis-free GWAS could only reveal the tip of the iceberg. Through the candidate gene approach, only very few findings could show replicable significant association with ADHD, as reported by meta-analysis studies for the dopaminergic, noradrenergic, and serotonergic genes. 210 211 Several GWAS have been conducted followed by meta-analysis, which again failed reaching genome-wide significant results. 212 213 214 215 216 217 218 219 220 221 222 223 224 However, recently, the first genome-wide significance has been reached in a GWAS meta-analysis consisting of over 20,000 ADHD patients and 35,000 controls. 225 Twelve independent loci were found to significantly associate with ADHD, including genes involved in neurodevelopmental processes, such as *FOX2* and *DUSP6* . 225 But even in these findings the effect sizes are rather small to be used for diagnostic tools. Therefore, polygenic risk score approaches have emerged as a possible tool to predict ADHD. 202 Yet this approach needs further investigation now that genome-wide significance has been reached by Demontis et al. 225 However, at this point, it is not yet possible to exclude that rare SNPs of strong effect may also be responsible (similar to breast cancer) for a small proportion of ADHD cases due to the heterogeneity of symptomatology, illness course, as well as biological marker distribution, as outlined above.

## Treatment

### Multimodal Treatment of ADHD

A variety of national and international guidelines on the assessment and management of ADHD have been published over the last 10 years, not only for clinicians but also for patients and caregivers. 96 97 226 227 228 All guidelines recommend a multimodal treatment approach in which psychoeducation forms a cornerstone of the treatment and should be offered to all of those receiving an ADHD diagnosis, as well as to their families and caregivers.

According to the NICE Guidelines, the first step is always a planning process for the multimodal treatment with respect to the psychological, behavioral, and occupational or educational needs of the child and his/her family. 97 This planning phase could be organized as a “round table” with the child, parents, and other caregivers. The following aspects should be taken into account: the severity of ADHD symptoms and impairment, the relative impact of other neurodevelopmental or mental health conditions and how these affect or may affect everyday life (including sleep). In addition, resilience and protective factors, as well as the goals of the child and family, should be considered in the intervention process. The participation of child and parents in the planning and treatment process is more centrally outlined in recent guidelines and is emphasized in detail for the different treatment steps (e.g., NICE and S3 Guidelines). 96 97 The participation process is not just a one-time dialogue but should rather continue throughout all steps of the treatment process. Benefits and harms of nonpharmacological and pharmacological treatments should be discussed carefully and on the basis of the latest evidence. Preferences and concerns, and the importance of adherence to treatment, should be discussed and taken into account within the treatment process. Patients and their families or caregivers should be reassured, as appropriate that they can revisit decisions about treatments.

Multimodal treatment approaches also advocate a systematic adaptive procedure that combines different treatment modules according to the needs and situation of the patient and family. This may, for instance, include a first stage in which parent counseling is initiated, a second-stage encompassing, for example, individual behavioral therapy for the child, while the parents participate in a parent training program in parallel, followed by a third stage in which stimulant medication is started, etc. 229 230 Environment-centered interventions aim at the counseling or training of parents or the instruction of teachers at school or preschool. Parent training programs may be administered individually or in groups and have shown positive effects on parenting skills, ADHD behavior, and comorbid conduct problems. 231 232 233 Family therapy for ADHD focuses on the ADHD family, with the ADHD patient being a part of the family system with dysfunctional interactional patterns. 234 School-based interventions may target (1) the conditions in the classroom, for example, by minimizing distractions; (2) the instruction of the teacher, for example, by suggesting more appropriate teaching methods or by promoting peer tutoring; or (3) the student, for example, by improving self-management and social skills, or by helping to cope with stigma. 235 236 237

### Pharmacological Approaches

#### Starting Medication

All medication for ADHD should only be initiated by a health care professional with training and expertise in diagnosing and managing ADHD. The expert should be familiar with the pharmacokinetic profiles and bioavailability of all the short- and long-acting preparations available for ADHD. The following parameters should be considered before first medication: medical history of the child but possibly also of the parents, current medication, height and weight, baseline pulse and blood pressure, a cardiovascular assessment, and an electrocardiogram if the treatment may affect the QT interval. A cardiology expert opinion should be sought before starting medication for ADHD if there is a history of congenital heart disease, previous cardiac surgery, or a history of sudden death in a first-degree relative under the age of 40 years, or if the blood pressure is consistently above the 95th centile for age and height for children and young people.

#### Age-Specific Needs

Treatment recommendations are often based on the specific needs of children, youth, or adults. 97 226 According to the NICE guidelines 97 and also pharmacological recommendations (e.g., Walitza and colleagues 238 239 ), a distinction should also be made between children under 5 years of age or preschool children, and school children. For the younger children (under 5 years of age), parent or career training programs and parent group training programs are always first-line treatments. Medication for children under 5 years with ADHD should only be given following a second specialist opinion from an ADHD service with expertise in managing ADHD in young children (ideally from a tertiary service). For children over 5 years of age, education and information about the causes and impact of ADHD and advice on parenting strategies should be offered, as well as liaison with school, college, or university if consent to do so is provided. 97 Children aged 5 years and over and young people should only receive medication if the ADHD symptoms are still causing a persistent significant impairment in at least one life domain after environmental modifications have been implemented and evaluated.

#### Selection of Pharmacotherapy

In Europe, methylphenidate either as short- or long-acting preparation is the first-line medication for ADHD across the life span. Second-line medications are lisdexamfetamine, atomoxetine, and guanfacine. A switch to lisdexamfetamine is only recommended if children have first undergone at least a 6-week trial of methylphenidate at an adequate dose and have not derived sufficient benefit in terms of reduced ADHD symptoms and associated impairment, or if patients experience adverse side effects. 238 The Canadian Guidelines (2018) recommend an individual treatment approach, which can start with different options, and if medication is to be used, long-acting formulations of psychostimulants or atomoxetine are always the first choice. 226 Comorbid disorders may necessitate adjustments to the treatment plan or alternative treatments.

According to the NICE guidelines, atomoxetine and guanfacine should only be offered if patients cannot tolerate methylphenidate or lisdexamfetamine or if their symptoms have not responded to separate 6-week trials of methylphenidate and lisdexamfetamine, having considered alternative preparations and adequate doses. 97

#### Evidence for ADHD Medications

In the first “gold standard” study comparing the different treatment approaches for ADHD alone and in combination (National Institute of Mental Health Collaborative Multimodal Treatment Study of Children with ADHD [MTA study]), the effects of both pharmacological therapy (methylphenidate and intensive counseling) and of multimodal therapy (methylphenidate and intensive behavioral therapy) were significantly more effective after 14 months than behavioral therapy alone or than the “standard” therapy (treatment as usual in the community) of the control group. The multimodal therapy was not significantly superior to pharmacological therapy alone, but did result in significant improvements in ADHD symptoms at a lower dosage of methylphenidate. 240 241 242 Since the MTA study, numerous studies have investigated methylphenidate, amphetamine, and nonstimulants like atomoxetine or α _2_ -adrenoceptor agonists, such as clonidine and guanfacine, regarding different aspects of effectiveness and tolerability.

The psychostimulants methylphenidate and amphetamine are the most effective agents for the treatment of core ADHD symptoms, with a favorable efficacy and adverse event profile. 243 244 245 Compared with methylphenidate and amphetamine, which both show immediate symptom reduction, the full effects of atomoxetine and guanfacine on reducing ADHD symptoms usually only unfold after some weeks of administration. Atomoxetine and guanfacine are not controlled substances, and are licensed in various European countries and in the United States for treatment of ADHD in children above the age of 6 years. Both have been shown to be effective in decreasing ADHD core symptoms with an effect size of around 0.7, which is somewhat lower than the effect size for methylphenidate, depending on the underlying studies (e.g., Sallee et al 246 ).

#### Management Strategies and Duration of Pharmacological Treatment

Following an adequate dosage of medication ( Table 1 ) and treatment response, medication for ADHD should be titrated to an optimized dosage with regard to the clinical efficacy, safety, and side effects, which should be continued for as long as it remains clinically necessary and effective. This should be reviewed at least annually, also with a planned “medication break” to decide whether there is a continuing need for care. 238 239 However, there is little available empirical evidence to guide clinicians on questions, such as the optimum duration of treatment and when it is appropriate to consider drug discontinuation. As ADHD can persist into adulthood, decisions on treatment discontinuation need to be taken on a case-by-case basis. 226

**Table 1:** Dosage recommendation for the most commonly used psychostimulants and other medications in the treatment of children and adolescents with ADHD

Generic name or trade name	Dosage (mg/kg body weight)	Total daily dosages (mg)	Number of doses per day
Methylphenidate immediate release	0.3– max. 1.0	5–40	1–3
Ritalin Medikinet		Max.: 60	
Methylphenidate sustained release	all preparations: 0.3–1.0		
Concerta		18–54 mg	1
Medikinet Retard		10–40 *	1
Ritalin LA		10–40 *	1
Amphetamine Liquid	0.1–0.5	2.5–20	1–2 (3)
		max. 40	
Lisdexamphetamine		30–70	1
Atomoxetine	0.5–0.8; max.1.2	If less than 70 kg:	1–2
Strattera		18–60	
		if more than 70 kg: 40–max. 100	
Guanfacine extended release	0.05–0.12	1–4	1
Intuniv			

* Without any reason the dose should not be increased above 60 mg.

Abbreviations: ADHD, attention deficit hyperactivity disorder; max. maximum.

Adapted from (1) Walitza S, Romanos M, Greenhill LL, Banaschewski T. Attention-Deficit/Hyperactivity Disorders. In: Gerlach M, Warnke A, Greenhill LL, eds. Psychiatric Drugs in Children and Adolescents. Wien: Springer; 2014:369–381 238 and (2) Walitza S, Gerlach M, Romanos M, Renner T. Psychostimulanzien und andere Arzneistoffe, die zur Behandlung der Aufmerksamkeitsdefizit-/Hyperaktivitätsstörung (ADHS) angewendet werden. In: Gerlach M, Mehler-Wex C, Walitza S, Warnke A, Wewetzer C, eds. Neuro-/Psychopharmaka im Kindes- und Jugendalter: Grundlagen und Therapie. Berlin, Heidelberg: Springer Berlin Heidelberg; 2016:289–331. 239

Among the most frequent side effects of psychostimulant therapy ( Table 2 ) are reduced appetite and sleep disturbances. 247 Appetite reduction following treatment initiation with an ADHD drug often attenuates with time. Reduced appetite at mealtimes can be avoided by taking the medication after meals rather than before. Should a clinically significant lack of appetite persist, dosage reduction (by one-fourth or half tablet of methylphenidate), discontinuation (rarely necessary), or switching to a different formulation or medication should be considered.

**Table 2:** Adverse drug reactions for psychostimulants and other medications in the treatment of children and adolescents with attention deficit hyperactivity disorder

Generic name	Adverse drug reaction
Methylphenidate (different methylphenidate formulations)	decreased appetite, insomnia or sleep disturbance, snoring, non-breathing or gasping while sleeping, sleepwalking, various sleep positions, enuresis, talking to sleep, number of hours of sleep, number of nocturnal movements, sleep quality, stomach upset, dizziness, headache, irritability, nausea, vomiting, tachycardia, increased blood pressure, moodiness, weight loss (higher dose), psychotic disorder, arrhythmias
Amphetamine (different formulations)	appetite suppression, weight loss, insomnia, stomach aches, headaches, irritability, dizziness, possible growth inhibition, exacerbation of psychosis, and tics, and possible increase in blood pressure and pulse
Atomoxetine	headache, nausea, abdominal pain, decreased appetite, moodiness and somnolence
Guanfacine	somnolence, sedation, headache, upper abdominal pain, and fatigue

Adapted from (1) Walitza S, Romanos M, Greenhill LL, Banaschewski T. Attention-Deficit/Hyperactivity Disorders. In: Gerlach M, Warnke A, Greenhill LL, eds. Psychiatric Drugs in Children and Adolescents. Wien: Springer; 2014:369–381 238 ; (2) Walitza S, Gerlach M, Romanos M, Renner T. Psychostimulanzien und andere Arzneistoffe, die zur Behandlung der Aufmerksamkeitsdefizit-/Hyperaktivitätsstörung (ADHS) angewendet werden. In: Gerlach M, Mehler-Wex C, Walitza S, Warnke A, Wewetzer C, eds. Neuro-/Psychopharmaka im Kindes- und Jugendalter: Grundlagen und Therapie. Berlin, Heidelberg: Springer Berlin Heidelberg; 2016:289–331 239 ; (3) Huang YS, Tsai MH. Long-term outcomes with medications for attention-deficit hyperactivity disorder: current status of knowledge. CNS Drugs 2011;25:539–554; (4) Storebo OJ, Pedersen N, Ramstad E et al. Methylphenidate for attention deficit hyperactivity disorder (ADHD) in children and adolescents - assessment of adverse events in non-randomized studies. Cochrane Database Syst Rev 2018;5:CD012069284; and (5) Wigal T, Greenhill L, Chuang S et al. Safety and tolerability of methylphenidate in preschool children with ADHD. J Am Acad Child Adolesc Psychiatry 2006;45:1294–1303.

### Nonpharmacological Treatments

#### Cognitive Behavioral Therapy

Cognitive behavioral therapy (CBT) is a form of behavioral intervention which aims at reducing ADHD behaviors or associated problems by enhancing positive behaviors and creating situations in which desired behaviors may occur. In the case of preschool and young school children, CBT focuses on parents and educators, who are instructed and trained to act according to CBT principles, while older children and adolescents may be trained directly to use more appropriate behavioral strategies. 248 CBT and its more specific forms (e.g., social skills training, training of planning and organizational skills, and self-management techniques) have positive effects on behavior, parenting skills, child–parent relationships, and certain daily living skills, 232 249 although effects on ADHD core symptoms are inconsistent and relatively low when only blinded assessments are considered. 250 A recent meta-analysis suggested that the combined treatment of medication with CBT is more efficacious than stimulant medication alone (with an estimated standardized mean difference of 0.5). 251

#### Neuropsychological Treatments

In cognitive training interventions, either PC-supported or in a manualized format, cognitive exercises that tap into cognitive domains, such as working memory or inhibitory control, are performed in a repetitive manner and with increasing difficulty. The evidence base for this type of intervention is poor according to recent studies (e.g., Bikic et al 252 ) and metastudies (e.g., Cortese et al 253 ). While some “near-transfer” improvements in neuropsychological tests tapping into the trained domain are probable, the evidence for “far transfer” to academic achievements or to the ADHD symptom level is weak. Most studies, however, used the same kind of cognitive training with all participants, irrespective of their actual individual cognitive difficulties. Moreover, they did not adhere to theoretically based training principles, which recommend domain-specific training for the functional improvement of a selective neuropsychological deficit. Possibly, future approaches that combine repetitive exercise and top-down strategy application may provide larger benefits for children with ADHD.

In neurofeedback training (NF), EEG activity measured by one or more electrodes applied to the head is transformed into a visual or acoustic signal and fed back online, for example, by a stimulus moving up and down. By steering the stimulus on the screen, the participant may gain control over his/her EEG activity. Many different training protocols have been applied to ADHD. Those which have received the best evaluation are the NF training of the θ/β frequency bands ratio (the goal is generally to decrease θ and to increase β frequencies) and the training of slow cortical potentials (learning to intentionally increase and decrease cortical excitability over short periods of time). However, “normalizing” an ADHD-specific deviant EEG pattern can no longer qualify as a meaningful goal, as no characteristic ADHD pattern seems to exist (Loo et al, 254 see neurophysiology section), although gaining control over one's brain activity and over attentional states continues to be a valid treatment goal. According to parent ratings, clinical improvements after NF are stronger and longer-lasting compared with other behavioral treatment methods, but teacher ratings usually fail to yield significant effects. 255 Recent research has focused on the specificity of treatment effects, defined as the association between the learned regulation of EEG activity and the behavioral outcome. 256 To date, there is no convincing evidence that the learned control over brain activity is responsible for the observed behavioral improvements. Instead, nonspecific treatment effects, such as improved self-efficacy, positive reinforcement, and learning to sit still, seem to contribute in large part to the positive clinical outcome.

Methodologically more sophisticated NF approaches, such as tomographic NF, 257 fMRI-NF, 258 or near-infrared spectroscopy feedback (feedback of hemoglobin oxygenation) 259 are still in the experimental stage.

#### Noninvasive Brain Stimulation

Repetitive transcranial magnetic stimulation (TMS) and transcranial direct current stimulation (tDCS) represent other potential means to modulate cortical activity. Therefore, these approaches may also be promising in terms of improving clinical and cognitive ADHD symptoms such as inattention and impulsiveness. 258 260 261 262 Based on a meta-analysis, Westwood et al 263 suggested that left and/or right prefrontal stimulation may improve performance in attention, inhibition and/or working memory tasks. However, these approaches are not yet recommended by therapy guidelines.

#### Alternative Nonpharmacological Treatment Methods

Mindfulness training, physical activity, and yoga seem to have positive effects on ADHD behavior, but for the time being, the scientific evidence is weak and these treatments are seen at best as complementary to other interventions. 264 265 266 267 268 Digital home treatment programs or support apps are currently being developed for ADHD patients or their parents 269 270 ; their usefulness or clinical validity still needs to be tested. Children and adolescents with ADHD often show a great affinity with digital media, which may improve compliance, but one has to take into account that the rate of problematic internet use and gaming is enhanced in youth with ADHD (estimated at 37% in ADHD vs. 12% in TD). 271 Free fatty acid supplementation has been described to bring about small but significant reductions in ADHD symptoms even with probably blinded assessments (standardized mean difference = 0.16). 250

## Long-Term Outcome

Follow-up studies have reported divergent results, with some reporting high rates of persistence until adulthood (up to 79%), 153 and others showing much higher rates of remission from childhood to adolescence (e.g., 45–55% of syndromal remissions). 272 273 274 Recent population-based studies from Brazil, the United Kingdom, and New Zealand have claimed that a large portion of de novo ADHD cases emerge at adult age, 275 276 277 but these results can probably be explained by methodological artifacts and missed subthreshold cases. 76 278 279 However, meta-analytic findings by Bonvicini et al 280 indicate that in part, different genes and polymorphisms seem to contribute to childhood ADHD and adulthood ADHD, lending some genetic plausibility to findings of a late manifestation of the disorder. According to the MTA study, the contribution of interventions administered during childhood to outcome in adulthood is negligible, but controlled intervention was limited to a relatively short period of time (14 months). 281 Neurobiologically, the course of ADHD may be explained by different models. 274 According to the first model, remission at adult age may be reduced to the normalization of brain functions through maturation. A second model explains remission through the recruitment of compensatory brain functions. The third model claims that brain function anomalies show life-long persistence, even though behavioral dysfunction may have remitted. 274 Possibly, all of these models, and probably additional ones too (see e.g., Doehnert et al 148 ), apply to different subgroups of patients or functions and may account for the divergent results in the literature.
